# Obstructive Sleep Apnea Among Obese Children in Tabuk City, Saudi Arabia

**DOI:** 10.7759/cureus.58714

**Published:** 2024-04-22

**Authors:** Laila AlBishi, Lulwah S Alkhuraisi, Mohammed M Alqahtani, Wejdan L Alatawi, Ahmed T Alghabban, Maram H Anazi, Hassan A Aljohani, Rammy A Asseiri

**Affiliations:** 1 Pediatrics, University of Tabuk, Tabuk, SAU

**Keywords:** saudi arabia, children, risk factors, obesity, obstructive sleep apnea (osa)

## Abstract

Introduction

Obstructive sleep apnea (OSA) is prevalent among children, impacting their well-being. Obesity and related morbidity may lead to serious health disorders. In obese children, OSA may be a risk factor for systemic diseases that negatively affect their quality of life. This study explored the correlation between obesity and OSA among children aged five to 14 years in Tabuk, Saudi Arabia.

Methods

This cross-sectional study employed an online questionnaire for the parents of 517 children, assessing sociodemographic variables, medical history, and OSA symptoms. The data analysis used Statistical Product and Service Solutions (SPSS; IBM SPSS Statistics for Windows, Armonk, NY) software, employing descriptive and inferential statistics.

Results

The children were predominantly male (281, 54.4%) and from Tabuk (405, 78.3%), with 158 (30.6%) classified as obese. Symptoms such as snoring (191, 36.9%), daytime fatigue (195, 37.7%), and impact on daily activities (79, 15.3%) were prevalent. OSA scores significantly correlated with BMI categories (p < 0.001), family history of OSA (p < 0.001), and medical conditions including diabetes, hypertension, and high cholesterol (p < 0.05). Correlations showed weak positive associations of age (ρ = 0.159) and height (ρ = 0.229) with OSA score, whereas a strong correlation existed between weight (ρ = 0.531) and OSA score (p < 0.001).

Conclusion

Obesity demonstrated a strong association with OSA severity among children in Tabuk. Higher BMI categories, a family history of OSA, and certain medical conditions correlated significantly with increased OSA scores. Although age and height displayed weaker associations, weight emerged as a major contributing factor to OSA severity. These findings emphasize the importance of addressing obesity in managing pediatric OSA, advocating for early interventions to mitigate its impact on children’s health and well-being.

## Introduction

An apnea is described as an airflow stoppage at the level of the mouth and nostrils that lasts at least 10 seconds. Three categories of apnea exist: obstructive sleep apnea (OSA), which occurs when the airways are obstructed; central sleep apnea, which is due to decreased or absent respiratory muscle activity; and mixed apnea, which starts as central apnea and then progresses to obstructive apnea [1].

OSA is a common disorder in children, brought on by recurring bouts of upper airway blockage while sleeping, resulting from increases in upper airway collapsibility. Underlying anatomical changes and problems with upper airway neuromuscular control, both of which are important in the pathophysiology of OSA, can make people more susceptible to collapsibility [2]. Inadequate air exchange during sleep results from aberrant narrowing in the nose, nasopharynx, oropharynx, or hypopharynx, causing clinical symptoms [3]. The symptoms vary with age and include morning headaches, snoring, nighttime waking, and excessive daytime tiredness [4].

OSA is a common disorder that affects 4-9% of the world’s population and people of all ages. Evidence suggests that the prevalence of OSA varies by ethnicity, being higher in African-American children than in white children [5]. How the disorder manifests is dependent on factors including weight, age, ethnicity, and gender.

Strong risk factors for sleep apnea include obesity, especially central obesity [6]. Obesity is defined as a BMI at or above the gender-specific 95th percentile according to the Centers for Disease Control and Prevention (CDC) BMI-for-age growth charts. According to data from the National Health and Nutrition Examination Survey (1976-1980 and 2003-2006), the prevalence of obesity increased from 5.0% to 12.4% in children aged 2-5 years, from 6.5% to 17.0% in those aged 6-11 years, and from 5.0% to 17.6% in those aged 12-19 years) [7]. In 2004, they stated that children with obesity and OSA face a “double challenge.”

A holistic approach to management for these children requires a clear understanding of how both problems interact. A study found that weight loss appears to confer benefits not only to OSA severity but also in mitigating cardiometabolic consequences of both OSA and obesity [8]. Unfortunately, weight loss through diet, exercise, and medication has been difficult to achieve and maintain. Bariatric surgery may be an alternative treatment for severe or complicated obesity; important and sometimes impressive changes have been noted in cardiovascular risk factors, metabolic markers, and OSA severity. Additionally, continuous positive airway pressure (CPAP) therapy may contribute to the weight loss process and, in and of itself, improve some of the metabolic abnormalities characteristic of OSA and obesity. However, treating OSA clearly cannot be limited to any single strategy. Rather, it requires a multidisciplinary approach, effective provider-patient communications, and systematic long-term follow-up to achieve effective and long-lasting therapeutic success. A study demonstrated that similar to adults, adolescents show an increased risk of obstructive sleep apnea syndrome (OSAS) in association with overweight and obesity [9].

For Caucasian children, overweight and obesity should be considered significant risks for OSAS among adolescents or from age 12 years, especially when combined with other established risk factors, including snoring and adenotonsillar hypertrophy [10]. This study aimed to assess the effect of obesity on the risk of OSA and determine the relationship between obesity and OSA, among children in Tabuk City, Saudi Arabia.

## Materials and methods

This cross-sectional study used an online structured questionnaire designed to collect data from participants. The target population was parents of children in Tabuk, Saudi Arabia, aged 5-14 years, with a BMI greater than or equal to the 95th percentile. A simple random sampling technique was used to collect the data. The sample size was calculated using the Raosoft calculator based on the population size of children aged 5-14 years in Tabuk (approximately 182,805). To obtain a 95% confidence interval and 5% margin of error, we needed to enroll at least 384 participants. The survey was conducted via a self-administered electronic Google Forms questionnaire. Individuals electronically provided their consent before participation. The general areas covered in the questionnaire were sociodemographic data, attitude, family history, and medical and surgical history. The questionnaire was designed in Arabic. The data were collected from July 2023 to December 2023.

The study maintained the participants' confidentiality. The investigators ensured the security of the data and informed the participants that it would not be used for any other purpose than this study. Personal data, such as names and contact information, were not included in the questionnaire.

The inclusion criteria were children aged 5-14 years, of any gender, who were overweight (85th-95th), obese (>95th), or extremely obese (>120th) percentiles compared to their peers. The exclusion criteria were older and younger children and those whose parents refused to participate in the study.

Statistical analysis was performed to assess the effect of obesity on OSA among children in Saudi Arabia. A structured questionnaire was designed to evaluate the presence and severity of OSA. It comprised five specific inquiries related to symptoms associated with OSA. Each question addressed specific symptoms: snoring, daytime tiredness, sleepiness, bedwetting, and the impact of OSA or obesity on daily activities. Participants responded to these questions on a scored scale ranging from “never” to “always”: never (0), sometimes (1), often (2), and always (3). To determine the OSA score for each participant, their scores for these five questions were totaled. The analysis involved both descriptive and inferential statistical tests. Simple frequencies and percentages of the categorical variables were calculated and tabulated. The Mann-Whitney U and Kruskal-Wallis tests were used to compare the OSA score among various qualitative factors. These non-parametric tests were chosen due to the non-normal distribution of the OSA score, calculated using the Shapiro-Wilk test (p < 0.05). Statistical significance was established at a p-value of 0.05 or less with a 95% confidence interval. All statistical calculations were performed using Statistical Product and Service Solutions (SPSS, version 27.0.1; IBM SPSS Statistics for Windows, Armonk, NY).

## Results

Among the 517 respondents, the mean age of the children was 10 ± 6 years. Table 1 presents the sociodemographic data of the participants. The gender distribution shows 236 (45.6%) girls and 281 (54.4%) boys. Most were from Tabuk (405, 78.3%), followed by Riyadh (31, 6.0%). Saudi nationals constituted 490 (94.8%) of the sample. Regarding the monthly family income, 289 (55.9%) had more than 10,000 SAR, 188 (36.4%) fell between 5,000 SAR and 10,000 SAR, and 40 (7.7%) had less than 5,000 SAR. According to BMI, 240 (46.4%) of participants exhibited normal weight, 95 (18.4%) were overweight, and 158 (30.6%) were obese. A family history of OSA was reported by 96 (18.6%) participants. The prevalence of specific diseases included 31 (6.0%) with diabetes, and medication use was reported by 62 (15.7%). The adenoidectomy and tonsillectomy rates were 38 (7.4%) and 61 (11.8%), respectively. Down syndrome was present in 7 (1.4%) participants. The mean values and standard deviations for age, weight, and height were 10 ± 6 years, 51.5 ± 108.0 kg, and 127.08 ± 30.68 cm, respectively.

**Table 1:** Descriptive statistics of sociodemographic data (n=517) SD: standard deviation; n: frequency of respondent; %: percentage

Variable	n (%)
Gender of the child	
Female	236 (45.6%)
Male	281 (54.4%)
Place of residence	
Madinah	20 (3.9%)
Makkah	16 (3.1%)
Riyadh	31 (6.0%)
Tabuk	405 (78.3%)
Others	45 (8.7%)
Nationality	
Saudi	490 (94.8%)
Non-Saudi	27 (5.2%)
Family income	
Less than 5,000 SAR	40 (7.7%)
Between 5,000-10,000 SAR	188 (36.4%)
More than 10,000 SAR	289 (55.9%)
Body mass index (BMI)	
< Normal weight (<5th)	24 (4.6%)
Normal weight (5th-85th)	240 (46.4%)
> Normal weight (85th-95th)	95 (18.4%)
Obesity (>95th)	92 (17.8%)
Severe obesity (>120th)	66 (12.8%)
Obesity	
Yes	158 (30.6%)
No	359 (69.4%)
Family history of obstructive sleep apnea (OSA)	
Yes	96 (18.6%)
No	421 (81.4%)
Medical history of the child	
Kidney disease	11 (2.1%)
Heart disease	12 (2.3%)
High cholesterol	16 (3.1%)
Hypertension	10 (1.9%)
Diabetes	31 (6.0%)
Other	133 (25.8)
No	304 (58.8)
If yes, is your child taking any medications?	62 (15.7%)
Has your child undergone adenoidectomy?	38 (7.4%)
Has your child underwent tonsillectomy?	61 (11.8%)
Does your child have Down syndrome?	7 (1.4%)
Child's age (Mean ± SD)	10 ± 6
The child's weight in kg (Mean ± SD)	51.5 ± 108.0
Child's height in cm (Mean ± SD)	127.08 ± 30.68

The responses related to children’s sleep and well-being questions are illustrated in Table 2. Regarding the question of whether the child had loud snoring, 191 (36.9%) responded “never,” 170 (32.9%) “sometimes,” 81 (15.7%) “almost” and 75 (14.5%) “always.” On the question of daytime fatigue, 95 (37.7%) reported “never,” 157 (30.4%) “sometimes,” 93 (18.0%) “almost always,” and 72 (13.9%) “always.” For the question about sleepiness or lethargy during the day, 195 (37.7%) reported “never,” 152 (29.4%) “sometimes,” 94 (18.2%) “almost always,” and 76 (14.7%) “always.” About bedwetting during the night, 336 (65.0%) reported “never,” 111 (21.5%) “sometimes,” 33 (6.4%) “almost always,” and 37 (7.2%) “always.” Lastly, regarding the impact of OSA or obesity on the child’s ability to perform daily activities, 279 (54.0%) reported “never,” 90 (17.4%) “sometimes,” 69 (13.3%) “almost always,” and 79 (15.3%) “always.”

**Table 2:** The severity of symptoms in children with obstructive sleep apnea (OSA)

Question	Always	Almost	Sometimes	Never
	n (%)	n (%)	n (%)	n (%)
Loudly snoring while sleeping?	75 (14.5%)	81 (15.7%)	170 (32.9%)	191 (36.9%)
Fatigue during the day?	72 (13.9%)	93 (18.0%)	157 (30.4%)	195 (37.7%)
Sleepiness or lethargy during the day?	76 (14.7%)	94 (18.2%)	152 (29.4%)	195 (37.7%)
Bedwetting during the night?	37 (7.2%)	33 (6.4%)	111 (21.5%)	336 (65.0%)
Can sleep apnea or obesity impact a child's daily activities?	79 (15.3%)	69 (13.3%)	90 (17.4%)	279 (54.0%)

Table 3 shows the comparison of OSA scores with various factors. As the BMI category increased, a notable shift in OSA scores was observed: the more severe the obesity, the higher the OSA score. This relationship was statistically significant (p < 0.001), indicating that higher BMI and the presence of obesity are strongly associated with increased OSA scores. Children with a family history of OSA tended to have higher OSA scores compared to those without such a history, and this difference was statistically significant (p < 0.001). Conditions such as kidney disease, high cholesterol, hypertension, and diabetes, as well as the use of medications for these conditions, were associated with higher OSA scores. The difference in scores between those with and without these conditions was statistically significant (p < 0.05). Factors such as gender, place of residence, nationality, family income, adenoidectomy, tonsillectomy, and Down syndrome did not impact the OSA score.

**Table 3:** Comparison of OSA score among children based on different sociodemographic characteristics IQR: interquartile range; *: Mann-Whitney U test; **: Kruskal-Wallis test; significant result in bold: p<0.05

Variable			OSA score	P value
			Median (IQR)	
Gender of the child	Female		4 (0-8)	0.608*
	Male		4 (1-7)	
Place of residence	Al Madina		(1-6)	0.370**
	Makkah		(2-7)	
	Riyadh		(0-5)	
	Tabuk		(0-8)	
	Others		4 (1-6)	
Nationality	Saudi		4 (1-7)	0.366*
	Non-Saudi		4 (3-7)	
Family income	Less than 5,000		(2-7)	0.094**
	Between 5,000-10,000		(1-8)	
	More than 10,000		3 (0-7)	
Body mass index (BMI)	< Normal weight		5 (6-3)	<0.001**
	Normal weight		1 (0-3)	
	> Normal weight		4 (3-7)	
	Obesity		7 (4-10)	
	Severe obesity		12 (9-15)	
Obesity	Yes		9 (5-12)	<0.001*
	No		2 (0-5)	
Family history of obstructive sleep apnea (OSA)	Yes		11 (7-14)	<0.001*
	No		3 (0-6)	
Medical history of the child	Kidney disease	Yes	8 (4-13)	0.021*
		No	4 (1-7)	
	Heart disease	Yes	7 (3-14)	0.067
		No	4 (1-7)	
	High cholesterol	Yes	13 (6-15)	<0.001*
		No	4 (1-7)	
	Hypertension	Yes	11 (8-15)	<0.001*
		No	4 (1-7)	
	Diabetes	Yes	13 (9-15)	<0.001*
		No	4 (0-7)	
If YES, is your child taking any medications?		Yes	11 (5-14)	<0.001*
		No	4 (1-7)	
A child underwent adenoidectomy?		Yes	4 (1-7)	0.400*
		No	4 (0-7)	
A child underwent tonsillectomy?		Yes	5 (3-7)	0.132*
		No	4 (0-7)	
A child with Down syndrome?		Yes	6 (3-13)	0.150*
		No	4 (1-7)	

Applying post hoc analysis (Mann-Whitney U test) to the significant relationships found with the Kruskal-Wallis test for BMI showed that the following comparisons had significantly different OSA scores: normal weight (5th-85th) vs. underweight (<5th), normal weight (5th-85th) vs. obesity (>95th); normal weight (5th-85th) vs. severe obesity (>120th), less than normal weight (<5th) vs. obesity (>95th); less than normal weight (<5th) vs. severe obesity (>120th), more than normal weight (85th-95th) vs. obesity (>95th); more than normal weight (85th-95th) vs. severe obesity (>120th); and obesity (>95th) vs. severe obesity (>120th).

The correlations between OSA scores and other quantitative data on the children in the study are demonstrated in Table 4. A weak positive correlation existed between age and OSA score (correlation coefficient, ρ = 0.159), signifying that as children aged, the tendency for OSA scores marginally increased. A robust and highly significant positive correlation emerged between weight and OSA score (ρ = 0.531, p < 0.001*). Furthermore, height exhibited a moderate positive correlation with OSA score (ρ = 0.229, p < 0.001*), implying that as children’s height increased, a concurrent tendency existed for OSA scores to be elevated. All these relationships were statistically significant.

**Table 4:** Correlation of OSA score with child’s age, weight, and height *: Spearman rho correlation, **: P value: <0.05 is considered significant

	OSA score		
	Correlation coefficient*		P value
The child's age	0.159		<0.001**
The child's weight in kg	0.531		<0.001**
The child's height in cm	0.229		<0.001**

## Discussion

The primary objective of this research was to investigate the correlation between obesity and OSA and to gain insights into the role of obesity as a potential risk factor for the occurrence and severity of OSA. Obesity may impact the severity of OSA by altering normal airway functioning because of excessive fat deposition around the pharynx, which reduces the size of the airway channel. Patients are referred for polysomnography (PSG) testing based on a confluence of symptoms and risk factors suggestive of OSA, such as daytime snoring, excessive daytime drowsiness, and obesity. Hence, the incidence of OSA cases among patients sent to a sleep clinic may not accurately represent the overall occurrence of the condition in the general community. According to one study, 84.7% of patients had snoring as a positive predictive value for OSA [10]. Another study indicated that snoring was observed as a symptom in all individuals who had episodes of apnea throughout their sleep [11]. Moreover, several studies have characterized obesity as a significant risk factor for OSA. Considering the prevalence of snoring and obesity in our study, we anticipated a significant occurrence of OSA.

An initial review of sleep disparities among children in Saudi Arabia showed a widespread pattern. According to the findings of that research, a notable 23% of children had a significant susceptibility to OSA [12]. This is less favorable than in several similar reports. A prevalence of 12.1% was seen among Chinese students aged 6-14 years who were diagnosed with OSA [13], whereas lower rates were shown among German schoolchildren (10.1%) [14] and Greek children (6.9%) [15]. The prevalence of habitual snoring among children in Saudi Arabia (15.9%) was greater compared to Chinese (7.2%) [16] and British (7.9%) students [17], but lower than the prevalence seen among Brazilian schoolchildren (27.6%) [18]. This broad spectrum of results may be attributed to diverse sample techniques, varying age demographics, and cultural influences.

The occurrence of bedwetting during the night, though less prevalent in this study’s sample than others, has been linked to OSA in children. Data have explored the association between nocturnal enuresis and OSA [15,19]. Our study found a lower frequency compared to other symptoms, suggesting potential variations in manifestation or individual differences within this cohort. Moreover, the impact of OSA on daily activities, as highlighted in this study, presents a considerable concern. Nearly half of the sampled children (49.9%) displayed a potential interference with daily routines due to OSA-related symptoms. This finding concurs with previous literature emphasizing the broader impact of OSA beyond nocturnal symptoms, affecting daytime functioning and overall quality of life [20].

Our study did not demonstrate a significant difference in OSA scores between genders, aligning with some existing literature. Although earlier studies have suggested varied prevalences between male and female patients, this study found comparable OSA scores regardless of gender. It also revealed no significant disparity in OSA scores concerning the place of residence or nationality. This contrasts with some prior research indicating geographic variations in OSA prevalence based on ethnicity or regional factors [9]. Although family income did not exhibit a statistically significant association with OSA scores, the study underlined crucial connections between medical history and OSA severity. Factors such as obesity, family history of OSA, comorbidities (including diabetes, hypertension, and high cholesterol), and medication intake were significantly correlated with higher (80%) OSA scores [21].

The observed weak positive correlation between age and OSA score (ρ = 0.159, p < 0.001*) suggests a subtle inclination for OSA scores to marginally increase with advancing age. This finding aligns with a previous study that suggested an age-related progression of OSA severity in children, possibly because of developmental changes in airway anatomy or neuromuscular control. Notably, a strong and highly significant positive correlation (ρ = 0.531, p < 0.001) emerged between weight in kilograms and OSA score. This robust association echoes the well-established link between obesity and OSA in pediatric populations [22]. Another study emphasized the strong correlation between increased body weight and the prevalence and severity of OSA in children, underscoring the importance of weight management in the clinical management of pediatric OSA [23]. Similarly, the moderate positive correlation (ρ = 0.229, p < 0.001) observed between height and OSA score implies a concurrent tendency for OSA scores to elevate with increased height. Although the relationship between height and OSA severity has received less attention, some studies have hinted at an association, possibly linked to changes in upper airway dimensions or anatomical factors [24].

Our study has several limitations. Its cross-sectional design limits establishing causality between obesity and OSA. Additionally, using an online questionnaire might have excluded participants without internet access, potentially skewing the results. Longitudinal studies are needed for a more definitive understanding. The sample’s geographical limitation to Tabuk might not fully represent Saudi Arabia’s diverse population. The lack of PSG or direct clinical assessments for diagnosing sleep apnea could also have led to misclassification or underestimation of OSA.

## Conclusions

Our study underscores the significant impact of obesity, familial history, and specific medical conditions on exacerbating OSA among children in Tabuk, Saudi Arabia. As BMI increased, OSA scores showed a marked escalation, emphasizing the significant association between obesity and increased OSA severity. Moreover, children with a family history of OSA or specific medical conditions including high cholesterol, hypertension, and diabetes exhibited elevated OSA scores. Understanding these connections is crucial for tailored interventions and preventative measures to mitigate the risks associated with OSA in pediatric populations.
